# From Laboratory Studies to Clinical Trials: Temozolomide Use in IDH-Mutant Gliomas

**DOI:** 10.3390/cells10051225

**Published:** 2021-05-17

**Authors:** Xueyuan Sun, Sevin Turcan

**Affiliations:** Neurology Clinic and National Center for Tumor Diseases, University Hospital Heidelberg, 69120 Heidelberg, Germany; xueyuan.sun@dkfz-heidelberg.de

**Keywords:** IDH-mutant glioma, TMZ, combination therapy

## Abstract

In this review, we discuss the use of the alkylating agent temozolomide (TMZ) in the treatment of IDH-mutant gliomas. We describe the challenges associated with TMZ in clinical (drug resistance and tumor recurrence) and preclinical settings (variabilities associated with in vitro models) in treating IDH-mutant glioma. Lastly, we summarize the emerging therapeutic targets that can potentially be used in combination with TMZ.

## 1. Introduction

Gliomas are the most common primary malignant tumors in the central nervous system. Grade 2 and 3 gliomas are referred to as lower grade gliomas (LGG) and harbor mutations in the isocitrate dehydrogenase (IDH) gene [1]. IDH-mutant gliomas have a slower growth rate and longer survival than IDH wild type (IDH-wt) tumors [1,2]. IDH-mutant gliomas are classified into two subgroups based on the presence (astrocytoma) or absence (oligodendroglioma) of chromosome arms 1p/19q [3] and histological criteria [4]. Recently, the European Association of Neuro-Oncology (EANO) stratified IDH-mutant gliomas into three WHO grades: oligodendroglioma, WHO grade 2 or 3; astrocytoma, WHO grade 2 or 3; astrocytoma, WHO grade 4 [5]. Although slower growing (at a rate of ~4–5 mm per year [6]), the majority of IDH-mutant LGGs eventually undergo malignant progression due to activation of the PI3K/mTOR pathway as a result of PTEN loss [7,8] or enhanced PDGF signaling [9]. Detailed molecular diagnostic markers, and other common molecular and pathway alterations in IDH-mutant gliomas are summarized in Table 1.

**Figure 1:**
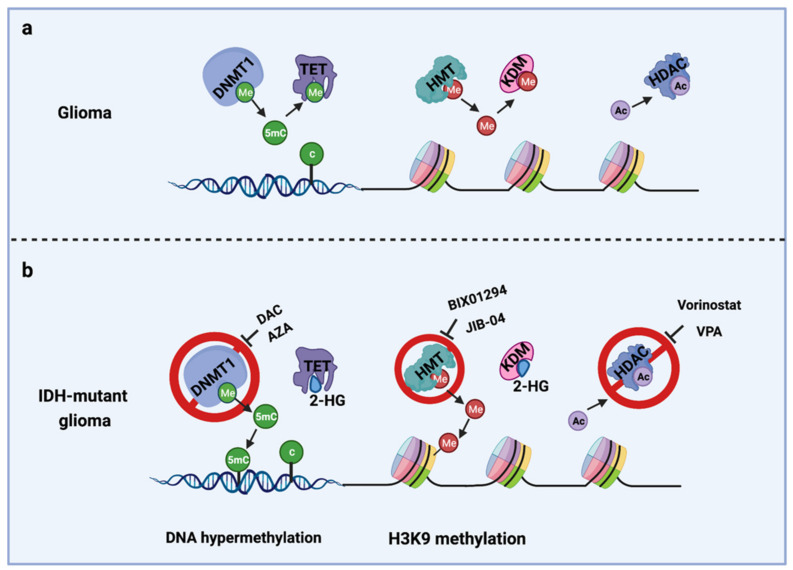
Epigenetic alterations induced by IDH mutations and potential drug targets for TMZ combination therapy. (**a**) Cellular epigenetic regulation without IDH mutation; (**b**) cellular epigenetic alterations in IDH-mutant gliomas. AZA, 5-azacitidine; DAC, Decitabine; DNMT1, DNA methyltransferase 1; HDAC, Histone deacetylase; 2-HG, D-(R)-2-hydroxyglutarate; HMT, Histone methyltransferase; KDM, Histone demethylase; TET, Ten-eleven translocation methylcytosine dioxygenases; VPA, Valproic acid; 5mC, 5-Mehylcytosine. Me: Methyl group; Ac, Acetyl group.

The IDH gene encodes the enzyme isocitrate dehydrogenase, which converts isocitrate to α-ketoglutarate (α-KG). α-KG an intermediate of the tricarboxylic acid (TCA) cycle that contributes to the production of NADPH. NADPH is necessary to reduce oxidized glutathione to glutathione, which directly neutralizes free radicals and reactive oxygen species (ROS). Overall, 65% of total NADPH in glioblastoma (GBM) is driven by the enzymatic activity of IDH, which is reduced to 38% when IDH is mutated [10]. There are three IDH isoforms, IDH1, IDH2, and IDH3, which are encoded by different genes. IDH1 is localized in the cytosol and peroxisomes, while IDH2 and IDH3 are located in the mitochondria. Among them, IDH1 is most frequently mutated in gliomas and harbors a monoallelic missense mutation of arginine to histidine at position 132 (IDH1^R132H^) at the catalytic site of the enzyme. IDH mutation produces a neomorphic enzyme that converts α-KG to D-(R)-2-hydroxyglutarate (2-HG), leading to the accumulation of 2-HG in the tumor [11]. The oncometabolite 2-HG is a competitive inhibitor of α-KG-dependent enzymes, including DNA demethylases (family of TET enzymes) and histone demethylases (family of Jumonji enzymes) [12,13]. This inhibition modifies the epigenetic status of histones and DNA, resulting in a plethora of cellular changes, including DNA hypermethylation [14] and altered histone methylation [15] (Figure 1). 

Treatment of LGGs includes surgery, radiation, and chemotherapy with either procarbazine/lomustine/vincristine (PCV) or temozolomide (TMZ). Here, we focus on the use of TMZ in IDH-mutant LGGs. First, we will present the effect of IDH mutation on cellular metabolism, epigenetic modifications, and the targeted therapies associated with these alterations. Second, we will discuss the use of TMZ in the treatment of IDH-mutant gliomas, including its toxicity, TMZ-associated molecular signature in tumor recurrence, and drug resistance, and discuss the synthetic lethality opportunities that emerge with TMZ treatment of IDH-mutant gliomas. Third, we will discuss the challenges of using TMZ to treat IDH-mutant gliomas in the preclinical setting, including non-consensus TMZ dosage and regimen, variable methods in measuring cell viability, and difficulties in culturing IDH-mutant glioma cell lines. To conclude the review, we will discuss targeted vaccine therapy that may facilitate the treatment of IDH-mutant gliomas. 

## 2. Cellular Alterations upon IDH Mutation and Targeted Therapies

**Figure 2:**
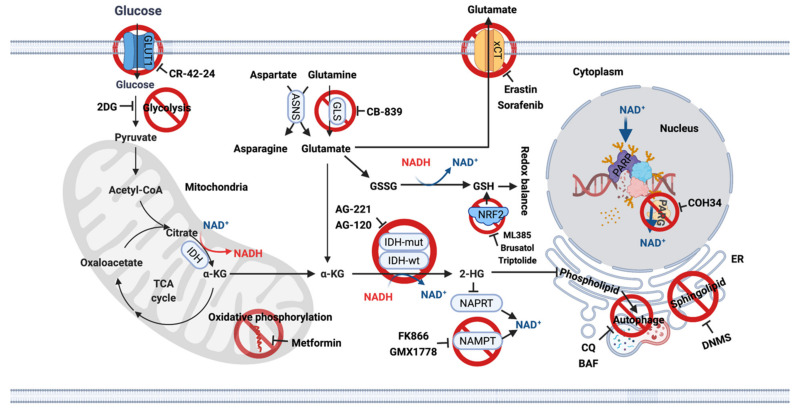
Metabolic vulnerabilities in IDH-mutant gliomas and potential targets for single treatment or combination treatment with TMZ. Actionable metabolic dependencies in IDH-mutant gliomas include interrupted redox balance, low basal levels of glutamate, NAD+ and lipid synthesis. ASNS, Asparagine synthetase; BAF, Bafilomycin A1; CQ, Chloroquine; 2DG, 2-Deoxy-D-glucose; DNMS, N,N-dimethylsphingosine; ER, Endoplasmic reticulum; GLS, Glutaminase; IDH, Isocitrate dehydrogenase; NAD+, Nicotinamide adenine dinucleotide; NAMPT, Nicotinamide phosphoribosyl transferase; NAPRT, Nicotinate phosphoribosyltransferase; NRF2, nuclear factor erythroid 2-related factor; PARP, Poly (ADP-ribose) polymerse; PARG, Poly (ADP-ribose) glycohydrolase.

Redox balance. IDH is a central enzyme of the TCA cycle; therefore, many studies have sought to understand the unique metabolic profiles reprogrammed by mutant IDH1 to therapeutically exploit these metabolic vulnerabilities [16] (Figure 2). IDH activity contributes to the majority of NADPH, which is used to maintain redox balance by converting oxidized glutathione to reduced glutathione. Therefore, IDH mutation leads to an increased oxidative burden, making interrupted redox balance a good therapeutic target. Synthesis of glutathione is mediated by the transcription factor nuclear factor erythroid 2-related factor (NRF2). Suppression of NRF2 by the natural compound brusatol [17], triptolide [18] resulted in profound tumor suppression in IDH-mutant xenografts, accompanied by overwhelming oxidative stress. 

Glutamate. Metabolomic analysis using glioma cell lines and surgical specimens indicated significantly reduced levels of glutamate in IDH-mutant gliomas [19,20,21,22], as glutamate used to produce α-KG is now used to synthesize 2-HG. IDH1 mutant tumor cells rely on glutaminase (GLS) activity to maintain α-KG homeostasis, making GLS inhibition a good therapeutic target [23,24]. However, preclinical studies indicated that the only GLS inhibitor CB-839 has only a moderate antiproliferative effect on IDH1-mutant cells, as cells compensate for reduced glutamate levels by upregulating asparagine synthetase (ASNS) [25], to generate glutamate via one of its amino acid precursors [26] (Figure 2).

Lipid synthesis. Altered lipid biosynthesis is another significant vulnerability of IDH-mutant gliomas. Biopsies from glioma patients showed lower phospholipid levels in IDH-mutant tumors compared with IDH-wt tumors. This effect was mediated by autophagic degradation of the endoplasmic reticulum (ER), termed ER-phagy [27]. Since ER is the site of phospholipid synthesis, late-stage autophagy inhibitors chloroquine (CQ) and bafilomycin A1 (BAF) restore phospholipid levels and trigger apoptosis in vitro and in vivo. This suggests that inhibition of ER-phagy may be a novel therapeutic option for IDH-mutant gliomas [27]. In a recent study, IDH1 mutation was found to increase monounsaturated fatty acids and their phospholipids [28]. The sphingolipid signaling pathway is also frequently activated in IDH-mutant gliomas, and inhibition of sphingosine kinase I (SphK1) with N,N-dimethylsphingosine (DNMS) specifically leads to cell death in IDH1-mutant gliomas [29]. All these studies provide therapeutic evidence for targeting lipid biosynthesis in IDH-mutant gliomas (Figure 2). 

NAD. Another extreme metabolic vulnerability is the low NAD+ levels in IDH-mutant cells, making further depletion of NAD+ a good therapeutic target [30]. Biosynthetic and consumptive processes maintain the intracellular NAD+ pool. Inhibition of the NAD+ synthesis enzyme nicotinamide phosphoribosyl transferase (NAMPT) with FK866 and GMX1778 [30] or activation of the NAD+ consuming enzyme sirtuin (SIRT) with SIRT1-activating compounds, or a combination thereof, reduce cellular NAD+ levels and inhibit the growth of IDH1-mutant tumor cells [31]. This suggests that targeting NAD+ metabolites may be a promising treatment option for these tumors. IDH-mutant gliomas are also sensitive to biguanides such as metformin, a metabolic inhibitor, which alters whole-body and cellular energy metabolism [32]. This treatment is currently being investigated in a phase Ib/II clinical trial to assess the efficacy of CQ in IDH-mutant gliomas (NCT02496741) [33,34]. This trial will provide direct evidence for targeting the metabolic rewiring for cancer therapy (Figure 2). 

Epigenetic modifications. IDH-mutant gliomas exhibit a cytosine-phosphate-guanine (CpG) island methylator phenotype (G-CIMP) [35] characterized by a genome-wide hypermethylation induced directly by mutant IDH1 [14] (Figure 1b). Several studies from us and others have shown that DNMT1 inhibitors, decitabine (DAC) [36,37] and azacitidine (AZA) [38], exerts an antiproliferative tumor effect in vitro and in vivo in IDH1-mutant gliomas. 

## 3. Temozolomide Treatment in IDH-Mutant Gliomas

Standard-of-care treatment for gliomas includes maximal surgical resection, possibly followed by radiotherapy (RT) and chemotherapy with PCV or TMZ. Several randomized clinical trials [39] investigating dosing (EORTC 22844 [40]) and timing (EORTC 22845 [41]) for RT in LGGs show that RT alone provides no significant benefit for overall survival. Similarly, TMZ alone showed no significant difference in progression-free survival in patients with LGGs compared with the efficacy of RT (EORTC 22033-26033 [42]). However, RT combined with TMZ or PCV resulted in an overall survival benefit in patients [43]. IDH-mutant oligodendrogliomas benefit from the addition of PCV to RT (RTOG 9802 [44,45], RTOG 9402 [46], EORTC 26951 [47], and NOA-04 [48]), while RT plus TMZ treatment shows more benefit in astrocytomas in clinical (EORTC 26053-22054 (CATNON) [49], RTOG 0424 [50]), and retrospective [51] studies. The interim analysis of the CATNON trial indicate a trend toward benefit with concurrent TMZ in IDH-mutant tumors, but not in IDH-wt gliomas. Thus, EANO recommends RT + TMZ for the treatment of newly diagnosed astrocytomas. For oligodendrogliomas, EANO recommends RT + PCV for initial treatment, and TMZ is only recommended for recurrent tumors not being pre-treated with TMZ [5].

Currently, there are no mature data comparing TMZ and PCV or their combination with radiation for LGGs. The ongoing clinical trial ALLIANCE-N0577-CODEL comparing RT + TMZ with RT + PCV for anaplastic oligodendrogliomas with 1p/19q co-deletion could potentially provide a more definitive comparison between the two regimens [52]. Both PCV and TMZ have been associated with grade 3 and 4 hematologic toxicities. Clinicians largely suggest TMZ to patients instead of PCV (>85%) [53], considering the relative difficulty of administering intravenous vincristine and the greater toxicity of PCV [54], whereas TMZ is easy to administer and generally well tolerated [43,55]. The ongoing phase III EORTC-1635-BTG (Wait or Treat?) is a randomized phase III trial comparing early adjuvant treatment with radiotherapy and adjuvant temozolomide to active surveillance in patients with resected IDH-mutant astrocytoma. Here, we summarize the current challenges related to TMZ in gliomas with a particular focus on IDH-mutant tumors.

### 3.1. Mechanisms of TMZ Toxicity

**Figure 3:**
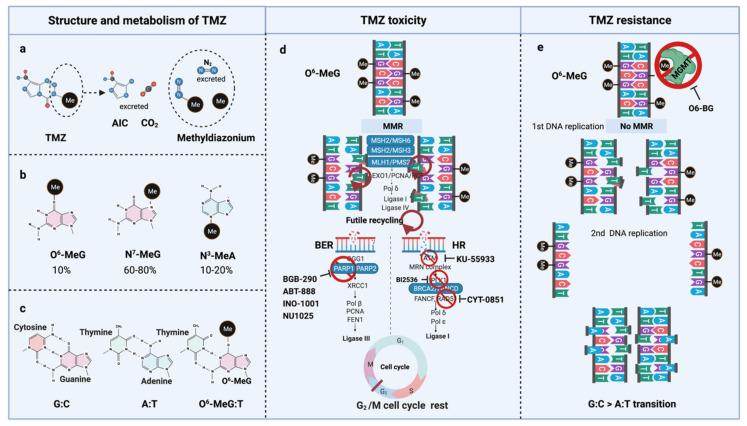
TMZ molecular structure, metabolism, toxicity, and resistance. (**a**) TMZ structure and metabolism, (**b**) DNA methylation upon TMZ, (**c**) DNA base mispair upon DNA methylation, (**d**) mechanism of TMZ toxicity with intact MMR, BER, and HR, (**e**) mechanism of TMZ resistance with functional MGMT and non-functional MMR. AIC, 4-Amino-5-imidazolecarboxamide; BER, Base-excision repair; HR, Homologous repair; MGMT, O^6^-methylguanine-DNA-methyltransferase; MMR, Mismatch repair; MLH, MutL homologue; MSH, MutS homologue; PMS, Post-meiotic segregation; TMZ, Temozolomide.

TMZ is administered orally in capsules at a dose of 150–200 mg/m^2^ for 5 out of 28 days for 6–12 cycles [5]. TMZ is a lipophilic DNA alkylating prodrug, and the cytotoxicity of TMZ is mediated by the addition of methyl groups to DNA. TMZ is an imidazotetrazine derivative of dacarbazine. Under neutral pH and aqueous conditions, it spontaneously decarboxylates to generate 5-(3-methyltriazen-1-yl)-imidazole-4-carboxamide (MTIC), which is further degraded to 4-amino-5-imidazole-carboxamide (AIC), and a highly reactive methyldiazonium ion that acts as a DNA methylating species [56] (Figure 3a). About 60–80% of methyl groups are added at DNA guanine residues (N^7^-MeG), 10–20% of the methyl groups are added at adenine (N^3^-MeA), and 10% of methyl groups at guanine (O^6^-MeG) [57] (Figure 3b). Single damaged bases, N^7^-MeG and N^3^-MeA, are readily removed by the rapid and efficient base excision repair (BER) system before replication. Therefore, the key toxic insult of TMZ is attributed to the O^6^-meG lesions [58,59].

O^6^-meG is considered the most genotoxic base modification due to the subsequent nucleotide mispairing with thymine (T) instead of cytosine (C) during DNA replication (Figure 3c). During replication, DNA polymerase inserts T opposite O^6^-meG. The mismatch repair (MMR) system can detect and repair these mismatches through the MutS and MutL complexes. The MutS recognition complex, including MutSα (an MSH2/MSH6 heterodimer) and MutSb (MSH2/ MSH3 heterodimer), identifies base–base mismatches and binds the O^6^-meG: T mismatch. Upon binding to the mismatch, the MutS complex recruits MutL (MLH1/PMS2 dimer) to the site of DNA damage. Together, these proteins excise a stretch of single-stranded DNA (ssDNA) containing the mispaired T, creating a gap in the DNA, while leaving the O^6^-meG adduct on the template strand intact [60]. DNA polymerase fills the gap by reinserting T opposite O^6^-meG, triggering another round of MMR which leads to repeated attempts to repair the same base T. This futile MMR cycling and accumulation of ssDNA gaps lead to successively longer DNA reinsertion and excision, which generates double strand breaks (DSBs) in subsequent rounds of replication and induce cell cycle arrest in G2/M phase, apoptosis and autophagy [61]. Thus, it needs two cell divisions for the emergence of TMZ cytotoxicity [62] (Figure 3d).

However, O^6^-meG lesions can be directly removed by O^6^-methylguanine DNA methyltransferase (MGMT) through covalent transfer, a process that effectively repairs the alteration prior to replication (Figure 3e). MGMT promoter methylation is a predictive biomarker of TMZ response in GBM. In general, the repair of O^6^-meG depends on the number of MGMT molecules per cell and the rate of MGMT regeneration [63]. In summary, the cytotoxicity from TMZ depends on low MGMT levels [64] and an intact MMR pathway [65].

### 3.2. Maintenance of TMZ Sensitivity

Efforts have focused on maintaining TMZ sensitivity by reducing MGMT levels or attenuating the activity of the BER and HR pathways for the duration of TMZ treatment to prevent resistance.

MGMT. O^6^–benzylguanine (O^6^–BG) is a potent inhibitor of the repair protein O^6^–alkylguanine–DNA alkyltransferase (AGT) that effectively inhibits MGMT activity by suicide inactivation. O^6^-BG binds and inactivates AGT, and until new AGT protein is synthesized, the cells have increased sensitivity to TMZ [66,67,68], leading to several clinical trials combining O^6^-BG and TMZ [67,69,70,71,72,73]. A phase II study showed that one-day dosing of O^6^-BG plus TMZ restored TMZ sensitivity in patients with TMZ-resistant anaplastic IDH-mutant gliomas [73] (Table 2).

MMR. MMR is regulated by multiple signaling pathways and responds to many stimuli [74]. Mutations in MMR genes and loss of MMR function are frequently detected in tumor samples [75]. Nevertheless, there is little research on how to maintain MMR integrity or directly increase MMR activity. One study showed that EGFRvIII expression and MAPK activation lead to increased MMR and therefore TMZ sensitivity [76], with direct clinical relevance that anti-EGFRvIII and anti-MAPK strategies should be used with caution in combination with TMZ.

BER. Many efforts have been made to target the downstream ssDNA repair and HR system to maintain TMZ sensitivity. One target from the base-excision repair (BER) pathway is poly (ADP-ribose) polymerase 1 (PARP1), which facilitates DNA repair by binding to single-strand breaks and recruiting DNA repair proteins to the site of damage [77]. PARP inhibitors (PARPi), INO-1001 [78], NU1025 [79], ABT-888 (veliparib) [80,81] and pamiparib [82], restored sensitivity in TMZ resistant glioma cells and xenografts. Several PARPi in combination with TMZ in GBM have been registered for clinical trials [83,84,85]. However, there are only two phase I clinical trials testing the efficacy of pamiparib (BGB-290) in combination with TMZ in newly diagnosed or recurrent IDH1/2-mutant gliomas (NCT03914742 and NCT03749187), and its clinical efficacy in IDH-mutant gliomas has yet to be demonstrated [86] (Table 2).

HR. One target from the HR pathway is the homologous recombinase RAD51. RAD51 is involved in DNA strand exchange between homologous DNA sequences. Exogenously expressed mutant IDH1 increases RAD51-driven HR and leads to increased TMZ resistance, and RAD51 knockdown increases the sensitivity of glioma cells to TMZ [87]. Several drug screens have identified inhibitors of RAD51 [88], but there is only one active clinical trial directly targeting RAD51 using a small molecule inhibitor CYT-0851 (NCT03997968). Another HR target is PLK1 (Polo-like kinase 1), which phosphorylates BRCA1 [89] and RAD51 [90] to promote homologous recombination. The combination of TMZ with a PLK1 inhibitor, BI2536, significantly suppressed the growth of IDH1-mutant glioma tumors [91], induced G2/M arrest, and suppressed cell proliferation and sphere formation [92]. Due to the toxicity of the small molecule inhibitor, knockdown of PLK1 using a small interfering RNA (siRNA) was combined with TMZ for glioma treatment, which showed enhanced anti-tumor activity both in vitro and in vivo [93]. However, limited success has been reported in preclinical studies with PLK1 inhibitors. ATM (ataxia telangiectasia mutated), a protein kinase that is a central mediator of responses to DNA double-strand breaks in cells [94], can also be targeted therapeutically. KU-55933, an ATM inhibitor, enhances the cytotoxic effects of TMZ in IDH1-mutant glioma cell lines [95].

Tumor Microtubes. Recently, there has been increasing evidence that tumor microtubes (TM) are an important mechanism of therapy resistance in gliomas. Gliomas interconnect and communicate through a network of TMs [96]. TM-connected glioma cells can self-repair, and are resistant to radiotherapy [96] and TMZ [97]. Inhibition of gap junctions with INI-0602 sensitizes primary GBM cells to TMZ [98], which shows the importance of pharmacological inhibition of the TM network. Furthermore, disrupting TM-based networks with meclofenamate (MFA) [99] sensitized primary glioblastoma cells to TMZ. The fact that TMs are more abundant in astrocytomas than in oligodendrogliomas [96], might explain why astrocytoma patients have a better response to TMZ than oligodendroglioma patients. A phase I/II trial evaluating safety as well as feasibility of a combined MFA-TMZ approach in relapsed MGMT-methylated glioblastoma (“MecMeth” EudraCT2021-000708-39) is being initiated in Germany [99].

### 3.3. TMZ-Associated Hypermutation

The first report investigating the effect of TMZ in the treatment of LGGs [100] showed that although most tumors exhibited initial chemosensitivity, the majority of tumors resumed progressive growth within a year of TMZ treatment, with astrocytomas (20/33) exhibiting a higher regrowth rate than oligodendrogliomas (5/30), implying that astrocytomas acquire accelerated TMZ resistance than oligodendrogliomas. Another long-term follow-up study showed that most oligodendrogliomas resumed growth within 3 years after TMZ [101].

TMZ resistance can be acquired either by elevated MGMT levels [102,103] or by mutations in the MMR machinery [104,105,106,107,108] that prevent futile MMR cycles at unrepaired O^6^-meG lesions. In the absence of MGMT-mediated repair in conjunction with deficient MMR, long-term TMZ treatment causes cells to accumulate G:C>A:T transitions throughout the genome, resulting in a hypermutator phenotype in recurrent tumors [63,109] (Figure 3e). Long-term TMZ treatment could also inactivate MMR pathway genes leading to hypermutation [109].

TMZ-induced hypermutation is observed more frequently in IDH-mutant than in IDH-wt gliomas [110,111]. However, it is not clear which subtype is more prone to develop the hypermutator phenotype. Reports from paired primary and TMZ-treated recurrent tumors show that astrocytomas have a higher rate of hypermutation [111,112], while data from random patient samples show that TMZ-induced hypermutation is more prevalent in oligodendrogliomas [109,110]. TMZ-induced hypermutation has been associated with a worse prognosis [112]; however, a larger cohort from the Glioma Longitudinal Analysis (GLASS) consortium shows no differences in overall survival between hypermutators and non-hypermutators [111].

Increased tumor mutation burden correlates with an elevated neoantigen load, indicating the potential to induce a durable response to immunotherapy [113]. However, current data show no discernible differences in the extent of immunoediting between initial and TMZ-treated relapsed hyper-mutated gliomas [111], and neoantigens from the recurrent hypermutators have relatively poor immunogenic qualities which may result in a weak anti-tumor T-cell response and likely a poor response to immunotherapy [109]. A current clinical trial is evaluating the immune-activating antibody pembrolizumab (MK-3475) in recurrent malignant gliomas that exhibit the hypermutator phenotype (NCT02658279).

### 3.4. TMZ-Induced Cellular Adaptations and Combination Therapy in IDH-Mutant Glioma

In addition to the known MGMT activity and DNA repair pathways in conferring TMZ resistance, efforts have been made to understand genetic, epigenetic, or metabolic adaptations following TMZ treatment. This knowledge could lead to synthetic lethal targeted strategies, with combinations of targeted therapies to circumvent some resistance mechanisms to delay or prevent malignant progression and recurrence [114,115]. Previous research has mainly focused on TMZ resistance in IDH-wt GBM [116,117,118], and here we summarize the current literatures on TMZ resistance mechanisms and therapeutic options in IDH-mutant gliomas.

#### 3.4.1. Genetic Mutations Associated with TMZ Treatment in IDH Mutant Glioma

Direct comparison of the genomic landscape of gliomas at initial diagnosis and recurrence has provided insight into the genomic alterations that may be associated with tumor recurrence after TMZ. Analysis of copy number alterations (CNAs) from primary IDH-mutant and IDH-wt gliomas of all grades revealed amplification of cyclins and cyclin-dependent kinase genes in IDH-mutant gliomas [119] (Table 1). A cohort of six pairs of initial untreated and TMZ treated recurrent IDH-mutant gliomas showed that recurrent tumors have driver mutations that activate retinoblastoma (Rb) and mammalian target of rapamycin (mTOR) pathways [112], which might drive malignant progression. The mTOR inhibitors such as rapamycin (RAPA) [120] have been reported to enhance TMZ-induced autophagic death of GBM cells and inhibition of the Akt-mTOR signaling pathway with amlexanox enhances TMZ-induced anti-tumor effects in preclinical GBM models [121]. An orally bioavailable dual PI3K/mTOR inhibitor, XL765 (voxtalisib), produced additive toxicity when combined with TMZ in genetically diverse GBM xenografts [122]. A phase I clinical trial (NCT00704080) demonstrated a favorable safety profile and a moderate inhibition of the PI3K/mTOR pathway in all glioma subtypes [123]. Sequential treatment of TMZ followed by PX-866, a PI3K inhibitor, inhibited TMZ-induced autophagy survival and enhanced apoptosis in GBM cells [124]. These findings suggest that PI3K/mTOR/Rb signaling pathways can be targeted separately or together to prevent tumor progression after TMZ treatment.

However, a study by the GLASS consortium comparing 23 pairs of untreated primary and TMZ-treated recurrent IDH-mutant gliomas [111] did not identify specific driver mutations associated with TMZ resistance. Across all cohorts, the hotspot IDH1^R132H^ mutation was not lost during progression and remained clonal in all progressed tumors [110], providing a good rationale for IDH^R132H^ vaccines for targeted therapies.

CRISPR-based screening enables sensitive detection of drug-gene interactions directly in human cells. Although no genome-wide CRISPR-Cas9 screen has been performed in IDH-mutant glioma models, results from GBM patient-derived lines [125] and GBM adherent lines [126] indicated that mismatch repair (MMR) and HR pathways are involved in TMZ resistance. In addition to the MMR pathways, an interesting molecular alteration detected in the human GBM cell line is NRF2 activation, and inhibition of NRF2 enhanced the anti-tumor effect of TMZ in glioma cells [127]. Since NRF2 is important for maintaining the redox balance in IDH-mutant gliomas and increasing ROS has been shown to augment chemosensitivity in IDH-mutant glioma [128,129], it is plausible that NRF2 inhibitors in combination with TMZ may be promising for the treatment of IDH-mutant gliomas.

Pathway analysis from RNA-seq data obtained from preclinical GBM models showed that epithelial–mesenchymal transition, Wnt signaling, and immune response were the most significantly activated pathways in TMZ-resistant cell lines [130]. In addition, negative regulation of telomere maintenance via telomerase was enriched in TMZ-sensitive glioma cell lines. A synergistic effect of a combination treatment of TMZ and a telomerase inhibitor, BIBR1532, was observed in in vitro models of GBM [130]. Whether telomerase inhibitors in combination with TMZ have an anti-tumor effect in IDH-mutant gliomas requires further investigation.

#### 3.4.2. Epigenetic Alterations upon TMZ Treatment

DNA methylation. Preclinical studies have shown that high TMZ concentration leads to a short-term increase in total 5-methylcytosine (hypermethylation), while repeated low TMZ doses lead to DNA hypomethylation [131]. This indirect effect on DNA methylation status may partly explain why 5-azacytidine (AZA) in combination with TMZ has a better anti-tumor effect in IDH1-mutant glioma patient-derived xenograft (PDX) models [132]. A phase I clinical trial of AZA combined with TMZ in patients with unresectable or metastatic soft tissue sarcoma or malignant mesothelioma shows that both drugs can be administered at their full dose without dose-limiting toxicities (NCT00629343) [133]. DAC, another DNA methyltransferase inhibitor, has been shown to potentiate TMZ treatment by enhancing the effects of DNA damage [134] and DNA mismatch repair [135] in GBM. A phase I/II clinical trial of the combination of DAC and TMZ in metastatic melanoma has shown that DAC can be safely added to extended-schedule TMZ and leads to improved response rates and progression-free survival (PFS) and overall survival (OS) rates in patients [136]. Due to the dose-dependent effect of TMZ on epigenetic modifications, further studies are needed to determine appropriate treatment regimens for the combination of TMZ and epigenetic therapy to achieve optimal clinical benefit.

Histone methylation. An inhibitor of histone methyltransferase (HMT) G9a, BIX01294, also exerted a synergistic effect with TMZ in GBM [137], possibly by enhancing the autophagy pathway. JIB-04, a novel inhibitor of Jumonji demethylases [138], synergized strongly with TMZ [139,140] in GBM in vitro and in vivo. Since IDH-mutant gliomas exhibit increased histone H3 lysine 9 (H3K9) methylation [141], the combination of G9a and TMZ may be a potential therapeutic target in IDH-mutant gliomas.

Histone acetylation. Several histone deacetylase (HDAC) inhibitors synergize with TMZ. For example, vorinostat [142] is well tolerated in combination with TMZ in GBM patients in a phase II trial (NCT00731731) [143] and are currently evaluated in combination with RT. Valproic acid (VPA) [144], another HDAC inhibitor with concurrent RT and TMZ are also well tolerated in GBM in a phase II study (NCT00302159) [145,146]. In a phase I clinical trial, a triple agent of dual epigenetic therapy, a combination of DAC, panobinostat (an HDAC inhibitor) and TMZ was well-tolerated, and its further efficacy is currently being evaluated in a phase II trial (NCT00925132) [147]. As epigenetic alterations may represent a global mechanism of resistance in cancer [148], preclinical experiments and clinical trials will clarify whether epigenetic therapy can act synergistically with TMZ in IDH-mutant gliomas.

Here, we summarize the current epigenetic drugs with TMZ combination therapy in Table 3.

#### 3.4.3. Metabolic Changes after TMZ Treatment

Glutamate. Previous studies have indicated that long-term TMZ treatment leads to changes in amino acid metabolism in preclinical models of oligodendroglioma [149]. Other reports identified that increased glutamate/glutamine/GLX (the sum of glutamate and glutamine) levels could be an early indication of response to TMZ treatment in IDH1-mutant gliomas [150,151]. IDH-mutant tumors have lower glutamate levels; thus, combination therapy of GLS inhibitor and TMZ may provide a greater benefit in IDH-mutant gliomas. Loss of xCT/SLC7A11, the glutamate exchanger that plays a role in ferroptosis, leads to increased vulnerability to TMZ [152,153]. These studies suggest that the effect of TMZ can be potentiated by ferroptosis inducing agents such as erastin and sorafenib. Another study showed that the addition of the glutaminase inhibitor CB-839 to TMZ significantly reduced aspartate and glutamate levels in an IDH-mutant patient-derived glioma xenograft model [154]. A phase I clinical study is currently evaluating the combination of CB-839, RT, and TMZ in IDH-mutated diffuse or anaplastic astrocytomas (NCT03528642) [155].

Phospholipid. The late-stage autophagy inhibitors chloroquine (CQ) and bafilomycin A1 (BAF) restore phospholipid levels and inhibit clonogenicity of IDH-mutant glioma cells. CQ enhances the cytotoxic effects of TMZ in GBM [156], and its clinical impact is being investigated in a phase I trial (NCT02378532). It is possible that the combination of CQ and TMZ disrupts the phospholipid balance and has greater synergistic effect in IDH-mutant gliomas.

NAD+. TMZ treatment leads to NAD+ consumption driven by PARP activation, as NAD+ is a known PARP cofactor. In IDH1-mutant cells with already low basal NAD+ levels, this surge in consumption leads to a further reduction in NAD+. Importantly, this metabolic imbalance introduces a window of hypervulnerability to NAD+ biosynthesis inhibitors [30]. Indeed, combined TMZ and NAMPT inhibition showed better efficacy in vivo than either agent alone [157]. Although the role of PARP in TMZ resistance is paradoxical as PARP needs to be inhibited to suppress its DNA repair function to maintain TMZ sensitivity but should be activated to drive NAD+ scarcity for its anti-tumor effect in IDH-mutant cells. The ongoing clinical trials of PARPi + TMZ in IDH-mutant glioma (NCT03914742, NCT03749187, NCT04394858, NCT01026493) [158] will give us a clear answer in the near future (Table 3). NAD+ is used for making poly (ADP-ribose) (PARylation) to recruit DNA repair factors [159]. PARylation is eventually degraded by PAR glycohydrolase (PARG), and NAD+ is regenerated. Therefore, combining TMZ with a PARG inhibitor COH34 leads to a scarcity of available NAD+, which is highly effective against IDH-mutant gliomas [160]. We expect PARG inhibitors with better toxicity profiles to be developed for preclinical and clinical trials in the near future.

Glucose. TMZ treatment has been shown to increase the expression of glucose transporters (GLUTs) [161,162], which triggers higher glycolytic activity and decreases the response to TMZ treatment, while inhibition of GLUT/SLC2A enhances the effect of TMZ [162]. Combination treatment with TMZ and paclitaxel (Taxol), a microtubule inhibitor, sensitized Taxol-resistant glioma cells via inhibition of glucose metabolism [163] and is currently in a phase II trial for the treatment of patients with metastatic melanoma (NCT01009515) [164]. Metformin, another metabolic inhibitor, alters both whole-body and cellular energy metabolism, and also shows a synergistic effect when combined with TMZ in GBM [165]. Trehalose, a natural disaccharide of glucose, combined with TMZ reduced clonogenicity and enhanced autophagic effects in melanoma cells [166]. Whether targeting glucose metabolism enhances the efficacy of TMZ in IDH-mutant gliomas requires further investigation.

A triple therapy combination with TMZ, CQ, and rapamycin decreased mitochondrial function and induced lysosome-dependent apoptotic cell death [167], suggesting that combinatorial targeting of metabolic and genetic alterations may be a good therapeutic option in cancer therapy in the future. Here, we summarize in Table 4 current vulnerable targets that could potentially be combined with TMZ in preclinical and clinical settings. Some of these have been tested only in GBM or other tumor entities but have the potential to be applicable to IDH-mutant gliomas as well.

## 4. Challenges of Using TMZ to Treat IDH-Mutant Glioma Cells in the Preclinical Setting

A recent review summarizes the four main limitations associated with the use of TMZ in preclinical models of GBM [168]: (1) the dosing and timing regimen of TMZ between clinical data and preclinical experimental design; (2) the dissolving agent, such as DMSO, and its low-dose toxicity; (3) cell lines that do not accurately represent the whole population; and (4) immunocompromised animal models with deficient immune systems. In this section, we focus on the use of TMZ in IDH-mutant glioma in vitro models, including TMZ administration, methods for determining cell viability, and difficulties in obtaining IDH-mutant glioma cell lines.

### 4.1. TMZ Treatment Dosage and Schedule

Human pharmacokinetic (PK) data determined exposure defined by the maximum plasma concentration (C_max_) and the integrated area under the plasma concentration-time curve (AUC), typically calculated from time zero to infinity, associated with the highest recommended dose of the drug [169]. With oral administration, 100% of the given TMZ dose enters the bloodstream. However, the site of action of TMZ requires its effective entry into the brain through the blood–brain barrier. There are no primary data showing how much of each metabolite eventually arrives at tumor cells in the human brain, with one report showing that the mean peak TMZ concentration in brain tissue was 0.6 ± 0.3 μg/mL, and the mean time to peak in the brain was 2.0 ± 0.8 h [170]. TMZ levels in the brain are only 20% of systemic drug levels, and it appears that the clinically relevant TMZ concentrations are around 5 µM [171]. Therefore, all in vitro studies should evaluate TMZ effects at low concentrations. There is no consensus on the concentrations and schedules used for TMZ in preclinical studies. To avoid the toxicity of DMSO [172,173] and maintain clinical relevance of TMZ dosage [174,175], we would recommend that TMZ dose should never be above 100 µM in vitro, and the dosing scheme should mimic that of a 5-day clinical schedule rather than a single high dose.

### 4.2. Cell Viability Assay In Vitro

Despite the wide range of cytotoxicity assays used in preclinical drug testing [176], the most commonly used assay methods in glioma research are the following: (1) dye exclusion assays using the trypan blue dye; (2) metabolism-based assays, including colorimetric assays that use MTT/MTS and luminometric assays that quantify ATP; (3) DNA synthesis proliferation assays using BrdU; (4) 3D soft agar growth assays.

The trypan blue dye exclusion assay [177] determines the number of viable cells based on the principle that live cells with intact membranes are impermeable to trypan blue. This method is used for suspension cells or trypsinized adherent cells, but it is associated with several pitfalls, including counting errors and reduced accuracy when viability is less than 80%. In addition, cell counting is usually performed with a hemocytometer, light microscope or automated cell counters, which makes simultaneous processing of a large number of samples difficult. On the other hand, MTT and MTS-based assays determine cell viability by the activity of mitochondrial enzymes such as succinate dehydrogenase [178]. Once MTT/MTS is reduced, the color changes from yellow to purple, which could be quantified by light absorbance at a specific wavelength, allowing the colorimetric measurement of cell viability using a spectrophotometer. Therefore, additional control experiments should be performed to reduce false-positive or false-negative results caused by background interference [179]. This method is applicable to both adherent and suspension cell lines and allows many assays to be performed simultaneously.

ATP-based assays are highly sensitive and quantify ATP content to determine the number of viable, metabolically active cells. In the presence of ATP, luciferin is oxidized to oxyluciferin, yielding a luminescent signal. Thus, the intensity of the luminescent signal is proportional to the concentration of ATP in active mitochondria. Because the IDH mutation alters multiple metabolic pathways, including NAD+ levels, redox balance, and oxidative phosphorylation, we should be extra cautious while interpreting the viability results from the metabolism-based assays, to ensure that we are only assessing the effect of drug treatment, and to rule out the effect of intrinsic metabolic alterations caused by 2-HG.

The BrdU (5-bromo-2′-deoxyuridine) cell proliferation assay monitors cell division by evaluating DNA synthesis through thymidine insertion. BrdU is a thymidine analog and can be incorporated into the newly synthesized DNA of replicating cells. BrdU levels can be detected by flow cytometry using a BrdU antibody conjugated with fluorochrome. Particular care must be taken with the incubation time for BrdU labeling as it is cell cycle dependent.

Soft agar colony formation assay measures the ability of single cells to form colonies in semi-solid soft agar medium in an anchorage-independent manner, which is closer to in vivo conditions. Depending on the cell lines and the number of cells seeded, the colony formation can take several weeks to months.

There is no perfect method to assess cell viability. While some assays can differentiate between dead and live cells, and others can just assess the relative performance of cells. To increase reliability of the results, researchers should use more than one assay method to better assess cell proliferation [180].

### 4.3. Cell Lines and PDX Models of IDH-Mutant Glioma

The IDH-mutant glioma cell lines used in preclinical research include four types: (1) genetically engineered lines overexpressing mutant IDH1 such as immortalized human astrocytes (IHA) [14,15,24,36,181], U87 [20,95,182,183], U251 [28,184,185], LN229 [95]; (2) patient-derived oligodendrogliomas: SF10417 [186], NCH612 [187], Hs683 [188], TS603 [28], BT088 and BT054 [189], BT237 and BT138 [190], BT054 [191], BT142 [192]; (3) patient-derived astrocytomas: SF10602 [186], NCH1681 and JHH273 [193], NCH551b and NCH620, and NCH645 and NCH3763 [194], MGG119 and MGG152 [30]; (4) non-glioma lines harboring endogenous IDH mutations, such as HT1080 [195].

Patient-derived IDH-mutant glioma cell lines are difficult to establish and propagate in vitro. One reason is the low incidence of IDH-mutant LGG patients at a given medical center, and another reason is that IDH-mutant tumor cells are difficult to culture [196] or to grow as xenografts [7,197] and can undergo loss of the IDH1 mutation in vitro. Therefore, regular cell line verification is required.

Due to rapid culturing and retention of the original genetic aberrations, some of the glioma cell lines have been used in several in vitro drug screens [194]. However, further evidence suggests that cell lines are poor representatives of the primary tumor isolated from patients in genetics, mRNA profiles, and protein expression [198]. Most experimental therapies with promising preclinical results using established cell lines have failed phase III clinical trials [199]. The development and propagation of mouse models and PDXs are more clinically representative but are time-consuming and costly [200].

Recently, 3D cultures of organoids have proven to be effective models. Organoids can be grown from patient-derived normal and tumor tissues [201] and eventually cultured in rotating bioreactors constituting various cell types, resembling defined brain regions [202]. Recently, Jacob et al. generated organoids for 96.4% IDH1-wt and 66.7% of IDH1-mutant gliomas [203]. Although the efficiency of generating organoids from IDH-mutant tumors remains to be optimized, IDH-mutant glioma organoids are likely to benefit personalized medicine and the development of individualized treatment for glioma patients [204].

## 5. Conclusions and Outlook

Here, we present the challenges and opportunities for treating IDH-mutant gliomas with the chemotherapy drug TMZ. We summarize the genetic, epigenetic, metabolic vulnerabilities that arise from IDH mutation and that could be exacerbated by treatment with TMZ. In the future, the fast-developing technologies, such as single-cell RNA-seq, or in vivo/in vitro shRNA and CRISPR screenings, will help us to identify more therapeutic targets in each specific molecular context, accelerating personalized medicine treatment in IDH-mutant gliomas. The recent success of the IDH1 mutation-specific vaccine in treating IDH1-mutated gliomas [205] may open new doors for the treatment of IDH-mutant gliomas. The integration of the immune system-assisted cancer cell elimination and chemotherapy-induced cancer cell death may be a new chapter for the treatment of patients with IDH-mutant gliomas.

## Figures and Tables

**Table 1:** Molecular diagnostic markers and common genetic alterations in IDH-mutant glioma.

Category	Alterations	Oligodendroglioma WHO Grade 2	Oligodendroglioma WHO Grade 3	Astrocytoma WHO Grade 2/3	Astrocytoma WHO Grade 4	
Diagnostic markers	IDH1 or IDH2 mutation	Present	Present	Present	Present	
	G-CIMP	Present	Present	Present	Present	
	ATRX			Inactivated	Inactivated	
	1p (FUBP1) / 19q (CIC) codeletion	Present	Present			
	TERT	Activated	Activated			
	9p21 (CDKN2A/B)				Inactivated	
	Necrosis and/or microvascular proliferation					Present
Other genomic alterations	TP53			Inactivated	Inactivated	
	Myc		Activated			
	TCF12		Inactivated			
	10q (PTEN/MGMT)				Inactivated	
Signaling pathways	Activation of PI3K signaling through loss of PTEN and gain of mTOR					
	Activation of cell cycle signaling through gain of CDK4, CDK6 and cyclin E2					

ATRX, Alpha-thalassemia/mental retardation, X-linked; CDKN2A/B, Cyclin-dependent kinase inhibitor 2A/B; CDK, Cyclin-dependent kinases; CIC, Capicua transcriptional repressor; FUBP1, Far upstream element binding protein 1; G-CIMP, cytosine-phosphate-guanine (CpG) island methylator phenotype; IDH, Isocitrate dehydrogenase; MGMT, Methylguanine-DNA-Methyltransferase; PI3K, Phosphoinositide 3-kinase; PTEN, Phosphatase and tensin homolog; TCF12, Transcription factor 12; TERT, Telomerase reverse transcriptase.

**Table 2:** Preclinical and clinical studies of combination therapy of TMZ with DNA damage repair pathway inhibitors.

Targeting DNA Damage Repair	Synergistic with TMZ	Preclinical Model	Clinical Trial	Arms	Tumor Type	Phase	Year
MGMTi	O6BG + TMZ	GBM PDX [68]; astrocytoma or GBM patient [67]	NCT00006474	O6-BG + TMZ	Astrocytoma	I	2001–2004
			NCT00389090	O6-BG + TMZ	Gliomas	II	(2006–2009)Terminated
			NCT00613093	O6-BG + TMZ	GBM	II	2002–2008
			NCT00275002	O6-BG + TMZ	Pediatrichigh-grade gliomas	II	2006–2010
PARPi	Olaparib + TMZ	U87-IDH mutant, U251-IDH mutant cell lines [81]	NCT03212742	Olaparib + TMZ + IMRT	GBM	I/IIa	2017–2022
			NCT04394858	Olaparib + TMZ	Pheochromocytoma and paraganglioma	II	2020–2023
	Veliparib (ABT-888)+TMZ	GBM BTICs and xenografts [80]	NCT01026493	ABT-888 + TMZ	Recurrent GBM	I/II	2010–2016
			NCT01514201	RT+ ABT-888 + TMZ	Children with newly diagnosed DIPG	I/II	2012–2018
			NCT02152982	veliparib + TMZ vs. placebo + TMZ	GBM	II/III	2014–2020
			NCT03581292	RT + TMZ + veliparib	GBM	II	2018–2024
	Pamiparib (BGB-290) + TMZ	GBM, GL261 murine glioma cells xenografts [82]	NCT03150862	BGB-290 + RT vs. BGB-290 + TMZ	GBM	1b/2	2017–2021
			NCT03914742	BGB-290 + TMZ	Recurrent IDH mutant glioma	I/II	2020–2023
			NCT03914742	BGB-290 + TMZ	IDH mutant glioma	I	2019–2027

BTICs, Brain tumor initiating cells; DIPG, Diffuse intrinsic pontine gliomas; GBM, Glioblastoma multiforme; MGMTi, Methylguanine-DNA-Methyltransferase inhibitor; PARPi, Poly (ADP-ribose) polymerase inhibitor; PDX, patient-derived xenograft; TMZ, Temozolomide.

**Table 3:** Preclinical and clinical studies of combination therapy of epigenetic drug with TMZ.

Epigenetic Target	Synergy with TMZ	Preclinical Model	Clinical Trial	Arms	Tumor Type	Phase	Year
DNMTi	AZA + TMZ	*IDH-mutant glioma* lines *BT142, JHH273* [132]	NCT00629343	AZA+TMZ	Soft tissue sarcoma or mesothelioma	I/II	2007–2014
	DAC + TMZ	GBM PDXs [134]	NCT00715793	DAC + TMZ	Metastatic melanoma	I/II	2008–2015
HDACi	Vorinostat + TMZ	GBM cell lines, glioma slice culture [142]	NCT00731731	RT + TMZ + Vorinostat	GBM	I	2009–2014
	VPA + TMZ	astrocytoma grade III and GBM cell lines [144]	NCT00302159	TMZ + VPA + RT	GBM	II	2006–2014
DNMTi + HDACi			NCT00925132	DAC + TMZ + Panobinostat	Melanoma	Ib/II	(2009–2016) Terminated

AZA, 5-azacitidine; DAC, Decitabine; DNMTi, DNA methyltransferase inhibitor; GBM, Glioblastoma multiforme; HDACi, Histone deacetylase inhibitor; RT, Radiotherapy; TMZ, Temozolomide; VPA, Valproic acid.

**Table 4:** Preclinical and clinical studies of TMZ combined with therapies targeting cancer metabolism

Metabolic Target	Combination Therapy	Preclinical Model	Clinical Trial	Arms	Tumor Type	Phase	Year
NAD+	NAMPT inhibitor + TMZ	IDH1 mutant glioma lines [30]	NCT00724841	GEM1777 + TMZ	Metastatic melanoma	I/II	2008–2010 (Terminated)
Glutamine	CB-839 + TMZ	GBM164 (IDH mutant) and GBM6 (IDH wt) PDX [154]	NCT03528642	CB-839 + RT + TMZ	Astrocytoma	1b	2018–2022
Oxidative phosphorylation	MET + TMZ	GBM PDX [165]	NCT01430351	MET + TMZ vs. mefloquine + TMZ vs. memantine + TMZ	GBM	I	2011–2022
Phospholipid	CQ + TMZ	GBM cell lines [156]	NCT02378532	CQ + RT + TMZ	GBM	I	2016–219
Multiple metabolites	Paclitaxel + TMZ	GBM cell lines [163]	NCT01009515	Carboplatin + Paclitaxel + TMZ	Metastatic melanoma	II	2009–2015 (Terminated)
	MET + CQ		NCT02496741	MET + CQ	IDH mutant glioma	1b	2015–2019

CQ, Chloroquine; MET, Metformin; NAMPT, Nicotinamide phosphoribosyltransferase; PDX, Patient-derived xenografts; RT, Radiotherapy; TMZ, Temozolomide.
